# Gold Nanospheres Dispersed Light Responsive Epoxy Vitrimers

**DOI:** 10.3390/polym10010065

**Published:** 2018-01-11

**Authors:** Zhenhua Wang, Zhen Li, Yen Wei, Yan Ji

**Affiliations:** The Key Laboratory of Bioorganic Phosphorus Chemistry & Chemical Biology (Ministry of Education), Department of Chemistry, Tsinghua University, Beijing 100084, China; zh-wang13@mails.tsinghua.edu.cn (Z.W.); chemlizhen@gmail.com (Z.L.)

**Keywords:** gold nanospheres, vitrimers, light responsive

## Abstract

Vitrimers represent a new class of smart materials. They are covalently crosslinked like thermosets, yet they can be reprocessed like thermoplastics. The underlying mechanism is the rapid exchange reactions which form new bonds while breaking the old ones. So far, heating is the most widely used stimulus to activate the exchange reaction. Compared to heating, light not only is much more convenient to achieve remote and regional control, but can also offer fast healing. Gold nanospheres are excellent photothermal agents, but they are difficult to disperse into vitrimers as they easily aggregate. In this paper, we use polydopamine to prepare gold nanospheres. The resultant polydopamine-coated gold nanospheres (GNS) can be well dispersed into epoxy vitrimers, endowing epoxy vitrimers with light responsivity. The composites can be reshaped permanently and temporarily with light at different intensity. Efficient surface patterning and healing are also demonstrated.

## 1. Introduction

From rockets to submarines, epoxy resins have been used in a vast array of applications as thermosets in modern society [1,2]. Classical epoxy resins become permanent cross-linked networks once cured. The network is unable to reshape any more. To address this problem, exchangeable links were added into traditional thermosets to form vitrimers [3,4,5,6,7,8]. Vitrimers can change their topology via dynamic exchange reactions under external stimulus. The first vitrimers were invented in 2011 [9] by adding a transesterification catalyst to an acid- or anhydride-cured epoxy network. The network performed like conventional epoxy thermosets at room temperature due to slow transesterification. However, once heated at high temperature, a rapid exchange reaction allowed the materials to be reshaped and recycled. Since then, various kinds of exchange reactions have been used to construct vitrimers [5,10,11,12,13,14,15,16,17]. 

Light-responsive vitrimers offer the material new properties that cannot be realized in heat-activated vitrimers. For example, as we have previously demonstrated [18,19], healing by light is much more effective than by heat. Meanwhile, light makes it possible to use lasers to reprocess vitrimers. New laser assembling techniques, such as welding, can be realized [18]. When using liquid crystalline elastomers as vitrimers, 3D dynamic structures can be easily fabricated, modified, and repaired [20,21]. However, in the previous research, all the light-responsive fillers have a wide range of absorption [18,19,22]. Compared with full-spectrum absorption agents, the absorption frequency of gold nanospheres can be selective and are adjustable by the structure [23]. When embedded into vitrimers, the matrix can only respond to selective wavelengths. This will make the material more stable when exposed to sunlight or light of other wavelengths. However, naked gold nanospheres aggregate significantly during the curing procedure of epoxy vitrimers. The aggregation results in the weakening of the plasmon resonance of the nanospheres. In this paper, we modified the gold nanospheres by polydopamine with the aim to reduce gold ions in situ and stabilize the coated spheres during high-temperature curing processes. 

## 2. Materials and Methods

### 2.1. Materials

Tetrachloroauric acid (J and K Chemical, Beijing, China, 99%), diglycidyl ether of bisphenol-A (J and K Chemical, Beijing, China, 99%), sebacic acid (Aladdin, Shanghai, China, ≥99.0%), and triazobicyclodecene (J and K Chemical, Beijing, China, 99%, L-DOPA (Aladdin, Shanghai, China, ≥99.0%) were used as purchased. 

### 2.2. Instrumental Analysis

Differential scanning calorimetry (DSC) was performed using a TA instruments Q2000 (TA Instruments, New Castle, DE, USA) operated at a scanning rate of 10 °C/min. Differential scanning calorimetry (DSC) was performed in the range of temperatures from 20.00 °C to 80.00 °C. The result of DSC was the second round of tests to eliminate effects of the thermal history. All of the experiments were in a nitrogen atmosphere. Thermogravimetric analysis (TGA) was conducted on a TA instrument Q50 (TA Instruments, New Castle, DE, USA) with a heating rate of 20 °C/min. Samples weighing about 5 mg were heated from 25 °C to 800 °C in air (60 mL/min) and N_2_ (60 mL/min) flow, both reactions taking N_2_ as the balance gas (40 mL/min). The stress-strain curves were measured by dynamic mechanical analysis (DMA) on a TA-Q800 (TA Instruments, New Castle, DE, USA). The infrared spectrum was recorded by an FTIR spectrometer (Perkin Elmer Spectrum 100 (PerkinElmer, Waltham, MA, USA)) with a universal ATR sampling accessory between 4000 cm^−1^ and 650 cm^−1^. The microscope used in this paper was a Nikon LV100POL (Nikon, Melville, NY, USA) microscope system. The dilatometry experiment was performed on a TA-Q800 DMA apparatus in the tension film geometry under the controlled force mode, and the sample was heated from 25 °C to 220 °C at a rate of 3 °C/min. Stress relaxation experiments were performed on a TA-Q800 DMA apparatus in the tension film geometry under the stress-relaxation mode. A strain was applied to the sample and maintained at 120 °C, 160 °C to relax stress in the following time.

### 2.3. Synthesis of Gold Nanospheres (GNS)

Twelve milliliters of L-DOPA solution (20 mmol) was stirred on a magnetic stirrer at room temperature. Ten milliliters of tetrachloroauric acid (HAuCl_4_, 10 mmol) solution was gradually dropped into the L-DOPA solution with stirring. The reaction was processed for 12 h at 25 °C, and the target polydopamine-coated gold nanospheres were obtained through centrifugation at 8000 r/min for 20 min.

### 2.4. Synthesis of GNS Vitrimers

Sebacic acid (0.202 g, 1 mmol) and diglycidyl ether of bisphenol-A (0.340 g, 1 mmol) were heated to melt at 160 °C. 0.0027 g (0.5 wt %) polydopamine-coated gold nanospheres were added under stirring. A total of 0.0139 g triazobicyclodecene was added quickly as a catalyst. Stirring of the mixture continued for 2 min, then the mixture was molded with Teflon under heat pressing at 180 °C for 4 h. 

## 3. Results and Discussion

### 3.1. Synthesis and Thermal Properties of GNS Vitrimers

**Figure 1:**
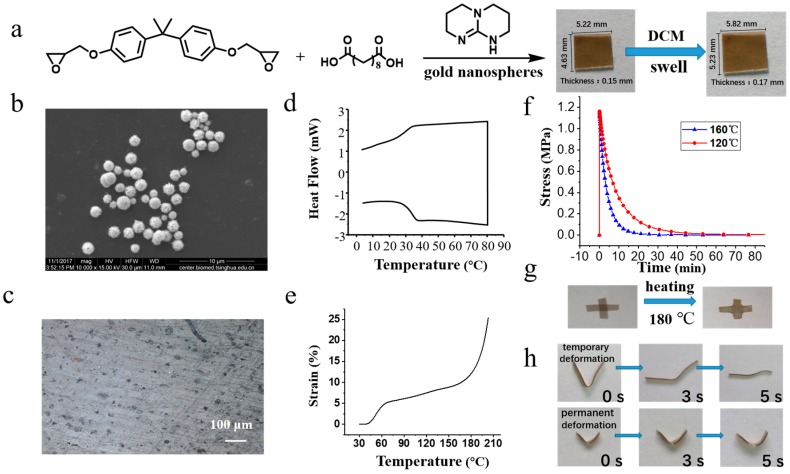
Synthesis and swell of GNS vitrimers (**a**); Image of old nanospheres by scanning electron microscope (**b**); Optical micrograph of the GNS vitrimers in transmission mode (**c**); DSC scan (rate of 5 °C/min) for of GNS vitrimers (**d**); Dilatometry test for GNS vitrimers (**e**); Stress-relaxation of GNS vitrimers at 120 °C and 160 °C (**f**); Welding experiment of two rectangular samples at 180 °C (**g**); Comparison of temporary deformation and permanent deformation once heated between *T*_g_ and *T*_v_ (**h**).

We synthesized the polydopamine-coated gold nanospheres by mixing L-DOPA solution with tetrachloroauric acid (HAuCl_4_) at room temperature. The resulting sphere diameters were around 1300 nm. The GNS vitrimers were prepared by adding polydopamine-coated gold nanospheres into sebacic acid-cured epoxy resin during curing (Figure 1b). Detailed synthesis of gold nanospheres and the sample preparation of GNS vitrimers are described above. The resultant vitrimers were swellable, but insoluble, in organic solvents. The test was conducted in dichloromethane. The sample was immersed in 5 mL (6.7 g) dichloromethane to test the insolubility. The original weight of the sample was 3.8 mg. This proved that the vitrimer’s volume reached 145% of its initial size after 30 min. In the following 3 h, the volume of sample remained constant and the sample did not resolve. After drying in a vacuum oven for 12 h, the sample lost 2.6% of its weight compared with the original weight. A solubility test above *T*_g_ was also processed in trichlorobenzene at 65 °C for 4 h. The volume of the vitrimer reached 147% (Figure S3) of its initial size after 4 h at 65 °C. The swelling experiment verified that the vitrimers were a cross-linked epoxy matrix. Fourier transform infrared spectroscopy (FT-IR) showed that the characteristic signal of epoxy groups (910 cm^–1^) had no apparent absorption peaks (SI). The GNS vitrimers appeared to be light brown due to the addition of polydopamine-coated gold spheres. There was no visible aggregation of nanospheres (Figure 1c). 

According to the differential scanning calorimetry (DSC) analysis, (Figure 1d) the glass transition of the GNS vitrimers was about 42 °C upon cooling. The transition temperature of the vitrimers changing from viscoelastic solid to viscoelastic liquid is described as *T*_v_. Above *T*_v_, transesterification between ester groups and hydroxyl groups in the network is accelerated, while the number of covalent bonds remains constant. According to dilatometry test (Figure 1e), when the sample was heated over 160 °C, the strain–temperature curve of the GNS vitrimers rose sharply. The network decomposed above 250 °C according to TGA in the Supplementary Materials. The increase of the slope should resulted from the acceleration of transesterification inside the vitrimers. For the stress relaxation experiment, stress comes from a preloaded strain. The dynamic bonds of the matrices will relax the stress by changing their topological structure. In this paper, we chose DMA to measure the dynamic characteristics of transesterification. As shown in Figure 1f, the stress relaxation of GNS vitrimers at 120 °C was much slower than that at 160 °C, reflecting the faster velocity of transesterification in GNS vitrimers at high temperature. The results of stress-relaxation also verified that the transesterification accelerated over 160 °C. The result of the welding test indicated that the GNS vitrimers could be reshaped permanently multiple times (Figure 1g). Once the material is heated between *T*_g_ and *T*_v_, the matrix will temporarily lose deformation because of entropic elasticity. However, permanent deformation will remain due to the changing of the topology. In Figure 1h, two bending samples were both heated at 75 °C. The temporary deformed sample recovered its original shape while the permanent deformed sample retained its bending structure.

### 3.2. Photothermal Effect and Applications of GNS Vitrimers

**Figure 2:**
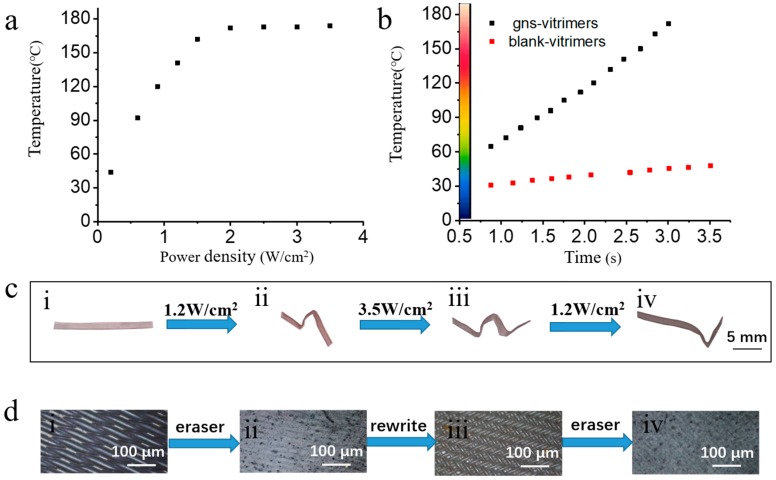
Local photothermal conversion of the GNS vitrimers at 3.5 W/cm^2^ (**a**); Comparison of GNS vitrimers and blank vitrimers upon different light intensity (**b**); Reshaping by light (**c**). Original shape (i). Reshaping the left section and central section at 1.2 W/cm^2^ (ii). Reshaping the right section at 3.5 W/cm^2^ to realize a “W” structure (iii). Reverting the “W” structure at 1.2 W/cm^2^ (iv). Surface patterning and repatterning (**d**).

The photothermal effect of gold nanospheres makes the local temperature of GNS vitrimers controllable. As shown in Figure 2a, the local temperature reached 185 °C in less than 4 s upon exposure to light (808 nm) of 3.5 W/cm^2^. As 185 °C is much higher than *T*_v_, it is possible to reshape and heal GNS vitrimers by light. To investigate the relationship between light intensity and temperature, an infrared camera was used to record the temperatures of both GNS vitrimers and blank vitrimers. Compared to blank vitrimers on the right, GNS vitrimers clearly show a higher temperature in Figure 2b. The difference of temperatures between GNS vitrimers and blank vitrimers increased as the laser power intensity increased. The result proved the dopamine-coated gold nanospheres made a major contribution to the light-thermal conversion in GNS vitrimers. 

The efficient light-thermal conversion of GNS vitrimers makes it possible to both temporarily and permanently reshape GNS vitrimers. Epoxy resins can be reshaped when heated above *T*_g_. However, such reshaping is temporary and the network will recover to its original shape once heated due to entropy elasticity. To fix a permanent new shape, vitrimers need a topology change above *T*_v_. As the temperature of GNS vitrimers increase rapidly under IR laser, the local temperature can be controlled via changing the light intensity. As illustrated in Figure 2c, a film was reshaped to a “W” by irradiation with an IR laser. The left and central sections were reshaped at 1.2 W/cm^2^ (Figure 2(cii)). At this intensity, the temperature was between *T*_g_ and *T*_v._ As a result, the shape was only temporarily fixed. The right section was irradiated at 3.5 W/cm^2^ to permanently lock the structure (Figure 2(ciii)). The left and central bend were flattened when irradiated at 1.2 W/cm^2^ again (Figure 2(civ)).

The light-reshaping process is not only possible in bulk vitrimers, but can also be done at the micrometer scale on the surface of GNS vitrimers. It is widely recognized that the surface structure can influence the surface properties of materials. This will be useful if the surface can be flexibly patterned. As shown in Figure 2d, a steel grid was pressed onto the film and then the whole set was exposed to light irradiation at 1.2 W/cm^2^ for 80 s. This led to a temporary pattern on the surface (Figure 2(di)). Then the film was irradiated at the same light intensity for another 80 s to erase the pattern. It was found that the surface of the film became smooth (Figure 2(dii)). Another steel grid was used to form a new pattern (Figure 2(diii)). That could also be erased again using the previous procedure (Figure 2(diii)). 

**Figure 3:**
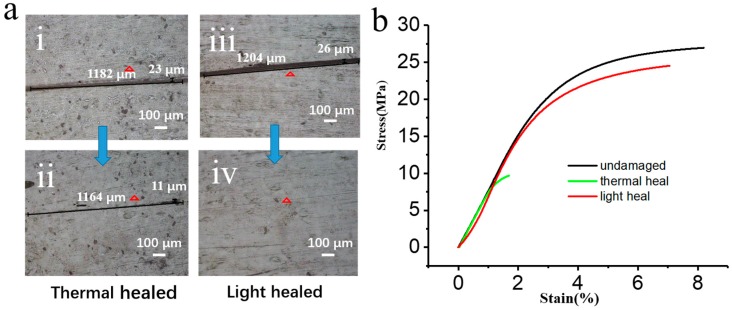
Healing of vitrimers (**a**), vitrimers before (i) and after (ii) heating for 10 min at 180 °C. Vitrimers before (iii) and after healing by light for 120 s (iv). Stress-strain curve of undamaged GNS vitrimers (black), thermal-healed vitrimers for 10 min (green), and light-healed vitrimers for 120 s (red) (**b**).

Traditionally, it is very difficult for thermosets to be healed without the help of glues or other materials once cured. However, GNS vitrimers can be activated by infrared laser in situ. Microcracks of vitrimers are able to be healed in a few seconds. As a demonstration, a film was cut with a razor and then exposed to an infrared laser of 3.5 W/cm^2^ for 120 s. As shown in Figure 3(aiii,aiv), the crack was healed completely. In contrast, as shown in Figure 3(ai,aii), the crack remained even after being heated at 180 °C for 10 min. According to tensile tests, the mechanical strength of the photo-healed sample was similar to the original, undamaged sample (Figure 3b). On the contrary, the damaged sample treated with heating was much weaker than the original one. All of the above experiments verify that light is a convenient and effective tool to heal GNS vitrimers.

## 4. Conclusions

Polydopamine-modified gold nanospheres enable photo-responsivity of epoxy vitrimers. The existence of the polydopamine coating on the gold nanospheres makes the gold spheres stable during the curing of epoxy vitrimers. Due to the strong photothermal effect of polydopamine-modified gold spheres, the transesterification reaction can be activated in vitrimers when illuminated. Therefore, the resultant composites can be reshaped and healed by light in a few seconds. In this way, surface patterning is also realizable. The method introduced here is simple and can generate other types of vitrimers. 

## Supplementary Materials

The following are available online at www.mdpi.com/2073-4360/10/1/65/s1, Figure S1: FTIR spectroscopy of vitrimers, Figure S2: Thermal gravimetric analysis, Figure S3: Swell test above *T*_g_ in trichlorobenzene.
