# Supplementary material for: Gold Nanospheres Dispersed Light Responsive Epoxy Vitrimers
Source: Polymers (Basel). 2018 Jan 11;10(1):65. doi: 10.3390/polym10010065 (PMC6414927; doi:10.3390/polym10010065)
Supplement: Supplementary file 1 [file polymers-10-00065-s001.pdf]

Supplementary Materials

Gold Nanospheres Dispersed Light Responsive Epoxy Vitrimers

Zhenhua Wang, Zhen Li, Yen Wei * and Yan Ji *

The Key Laboratory of Bioorganic Phosphorus Chemistry & Chemical Biology (Ministry of Education), Department of Chemistry, Tsinghua University, Beijing 100084, China; zh-wang13@mails.tsinghua.edu.cn (Z.W.); chemlizhen@gmail.com (Z.L.)

* Correspondence: weiyen@mail.tsinghua.edu.cn (Y.W.); jiyan@mail.tsinghua.edu.cn (Y.J.); Tel.: +86-(10)-6277-2674 (Y.W.); Tel.: +86-(10)-6278-7816 (Y.J.)

Supporting Data

FTIR Spectroscopy of Vitrimers

FTIR spectroscopy below demonstrates the conversion of epoxy groups because of the weak absorption at 910 cm^{-1}. The absorption peaks at 3442 cm^{-1} and 1734 cm^{-1} verify the hydroxyl groups and ester groups of the vitrimers.

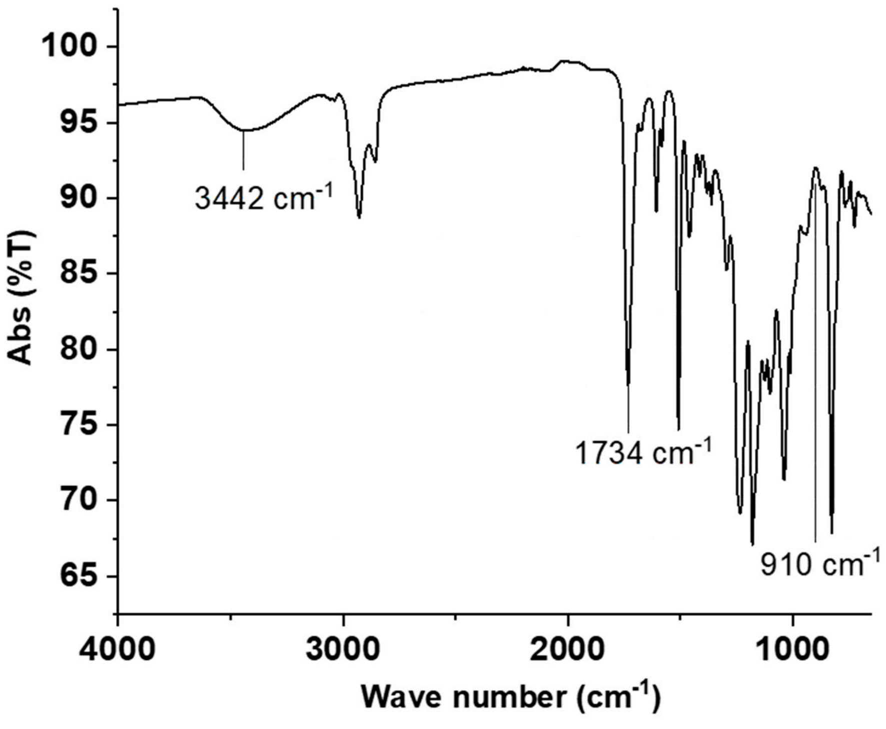

Figure S1. FTIR spectroscopy of GNS vitrimers.

Thermal Gravimetric Analysis (TGA)

Thermal gravimetric analysis proves the materials decompose at 310 ℃ in both air and N_2 conditions. The GNS vitrimers are stable below 200 ℃, and the sharp rise of the strain–temperature curve cannot be the result of the decomposion of vitrimers.

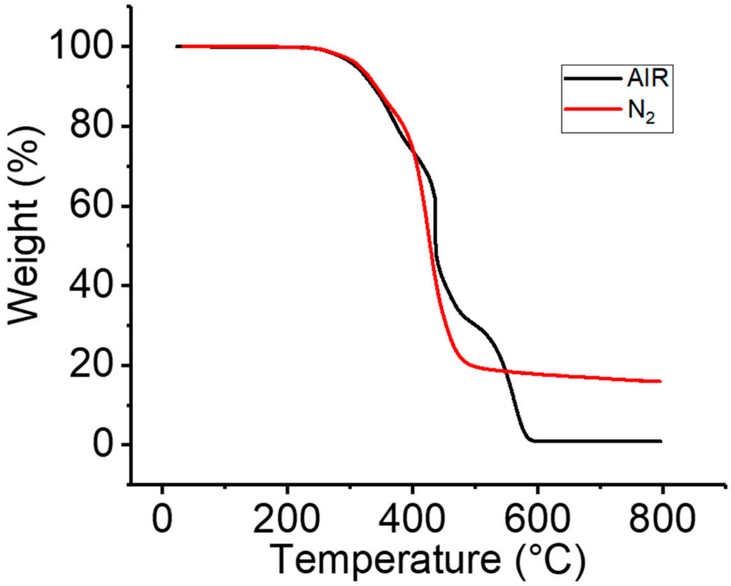

Figure S2. Thermal gravimetric analysis of the materials in both air and N_2 conditions.

Swell Test above T_g in Trichlorobenzene

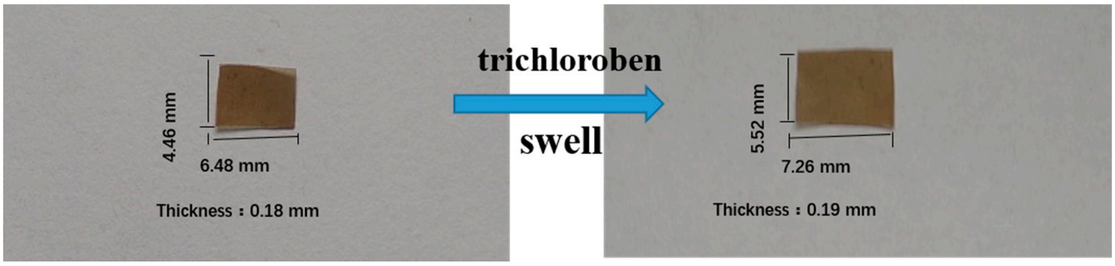

Figure S3. Swell test above T_g in trichlorobenzene.
